# Magnetic-Field-Assisted Scratching Process of Single-Crystal Copper

**DOI:** 10.3390/mi14122255

**Published:** 2023-12-18

**Authors:** Xian Wu, Kechuang Zhang, Ke Sun, Feng Jiang, Jianyun Shen, Hongyou Li, Lizhi Gu

**Affiliations:** 1 College of Mechanical Engineering and Automation, Huaqiao University, Xiamen 361021, China; 15993121246@163.com (K.Z.); wx19890909@126.com (K.S.); jianyun@hqu.edu.cn (J.S.); lihongy@hqu.edu.cn (H.L.); 2 Institute of Manufacturing Engineering, Huaqiao University, Xiamen 361021, China; jiangfeng@hqu.edu.cn; 3 Fujian University Key Laboratory of Virtual Manufacturing Technology, Quanzhou University of Information Engineering, Quanzhou 362000, China; gulizhi888@163.com

**Keywords:** metal cutting process, single-crystal copper, magnetic field, friction coefficient

## Abstract

Energy-field-assisted cutting exhibits excellent ability to reduce cutting force and improve machining quality. In this study, a magnetic field was applied in an innovative way to aid in the cutting process, and magnetic-field-assisted scratching experiments of single-crystal copper were carried out. It was found that magnetic-field-assisted scratching increased the actual scratching force due to the additional Lorentz force in the cutting process. However, the friction coefficient of the magnetic-field-assisted scratching was reduced by 19.4% due to the tribological modification effect on tool/chip contact. Meanwhile, magnetic-field-assisted scratching was conducive to decreasing the degree of chip deformation, reducing microburrs on the machined surface, and obtaining a surface roughness reduction of an average of 26.8%. The possible reason for this effect was that the presence of a magnetic field in the cutting process promoted the dislocation slip of metal materials. The results indicated that magnetic-field-assisted cutting improves the machinability in the metal cutting process.

## 1. Introduction

Continuous improvements in machining accuracy and surface quality represent a common focus in precision and ultra-precision cutting. Conventional cutting processes display their strengths in terms of machining accuracy and surface quality [1,2,3]. To further increase their ultimate machining ability, energy-field-assisted cutting methods, such as laser and ultrasonic vibration energy fields, have been proposed by scholars [4,5,6]. In the laser-assisted cutting process, the laser beam is focused on the local surface position to be machined to soften the workpiece material, and the cutting tool removes the softened material after the hardness decreases and the plasticity increases. It has been validated that this method can effectively reduce the cutting force, extend tool life, and improve the surface quality, especially when cutting materials with high hardness [7,8]. You et al. [9,10] proposed a laser-assisted turning method with in-process heating, in which the laser beam is directly guided on the workpiece surface that is being cut using a transparent diamond tool; this method has been successfully applied in the ultra-precision cutting of tungsten carbide and silicon freeform surfaces with nanoscale surface roughness. Wu et al. [11] proposed a laser oxidation-assisted cutting method to machine cemented carbide, and they found it could significantly reduce the cutting force. In the ultrasonic-vibration-assisted cutting process, the tool and workpiece periodically separate. The continuous cutting process becomes an intermittent cutting process and helps to reduce both the cutting force and tool wear [12,13]. The ultrasonic vibration equipment and ultrasonic-vibration-assisted cutting mechanisms and equipment include one-dimensional vibrations and multidimensional vibrations, and they have been widely studied by scholars worldwide. At present, the ultrasonic-vibration-assisted cutting method has been widely used in the turning, milling, and drilling of various workpiece materials, such as metal, ceramics, and composite materials [14,15,16,17,18]. In addition to the above energy fields, the flame, chemical, and ionic energy fields have also been studied to aid in the cutting process of various materials.

A magnetic field improves interface tribological characteristics and reduces the friction coefficient and adhesive wear [19,20]. A long time ago, Muju et al. [20] performed a friction test both with and without a magnetic field action, and they found that the presence of an external magnetic field could reduce the adhesive wear between mild steel and brass materials. Since this study, many scholars have studied the changes in the interfacial friction characteristics between various materials, such as carbon steel, nickel, and graphite, under the presence of an external magnetic field and have found that the effect of a magnetic field can effectively reduce interfacial wear. According to these studies, in addition to laser and vibration energy fields, a magnetic field has been introduced into the cutting process based on the magnetic antifriction effect [21,22]. Mansori et al. [23,24,25] first tried to install conductive coils around a high-speed steel tool or ferrous metal workpiece to perform magnetic-field-assisted turning, where the generated magnetic induction line was vertical or parallel to the workpiece. They found that the chip deformation coefficient, shear angle, and friction coefficient changed and a long tool life was obtained with the presence of a magnetic field in the cutting process. Subsequently, Mkaddem et al. [26] performed further studies on the magnetic-field-assisted cutting process and found that with an increase in magnetic field intensity in the cutting process, the shear angle increased, the contact length and friction coefficient between the tool/chip interface decreased, and the sawtooth degree of chips was also reduced. They pointed out that the presence of a magnetic field could promote dislocation slip and increase material plasticity during the cutting process, making the cutting process smoother. Dehghani et al. [27] studied the related cutting mechanisms and machinability in the magnetic-field-assisted cutting of ferromagnetic steel materials with good magnetism. They designed an L-shaped fixture with an excitation coil to generate a magnetic field perpendicular to the axis of the workpiece. The results showed that the presence of the magnetic field during the cutting process could decrease the cutting vibration and change the chip shape. Yip et al. [28,29,30] synthetically studied the magnetic-field-assisted single-point diamond turning of titanium alloy material, which presented weak magnetism. They pointed out that in the presence of a magnetic field, paramagnetic particles were generated along the direction of the magnetic field and gathered at the tool/chip interface, which improved the thermal conductivity of the titanium alloy and reduced the tool wear. At the same time, when the titanium alloy workpiece moved in an alternating magnetic field, the generated self-excited magnetic field suppressed the cutting vibration and improved the cutting stability. These studies all reported that magnetic-field-assisted cutting can improve machining quality to a certain degree, such through machining vibration and tool wear reduction, as well as thermal conductivity improvement. However, the workpiece objects in the above studies were all magnetic materials, and thus the machinability of magnetic-field-assisted cutting on nonmagnetic materials is still unknown.

In this study, to explore the machinability of magnetic-field-assisted cutting of single-crystal copper, without magnetism, magnetic-field-assisted scratching experiments were carried out. The effect of a magnetic field on the cutting process was studied. The scratching force, friction coefficient, chip morphology, and surface quality were analyzed and compared to those of conventional cutting. The research results are helpful in terms of exploring the application of the magnetic-field-assisted cutting process.

## 2. Experimental Procedures

**Figure 1:**
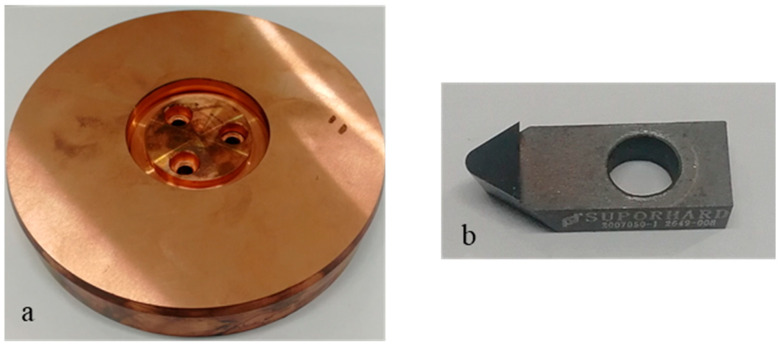
Single-crystal copper workpiece (**a**) and PCD tool (**b**).

The scratching process is approximate to the ultra-precision cutting process and often applied to investigate the related cutting mechanisms. Single-crystal copper produced by single-crystal continuous casting technology was utilized as workpiece material in the scratching test, as exhibited in Figure 1a. The top surface of a single-crystal copper workpiece was the crystal face (111), which presented poor machinability in this single-crystal material. The single-crystal copper workpiece was preprocessed into a cylindrical shape with a diameter of 148 mm and a height of 24 mm. The top surface was preprocessed into a surface roughness of less than 10 nm before the scratching experiments. The scratching test was performed along the radial direction on the top surface. A polycrystalline diamond (PCD) tool, which is widely used in the processing of nonferrous metal materials, was employed as the scratching tool in this study, as exhibited in Figure 1b. In terms of tool geometry parameters, the tip radius was about 1 mm, edge radius was about 5 μm, and rake and clearance angles were, respectively, 0° and 5°.

**Figure 2:**
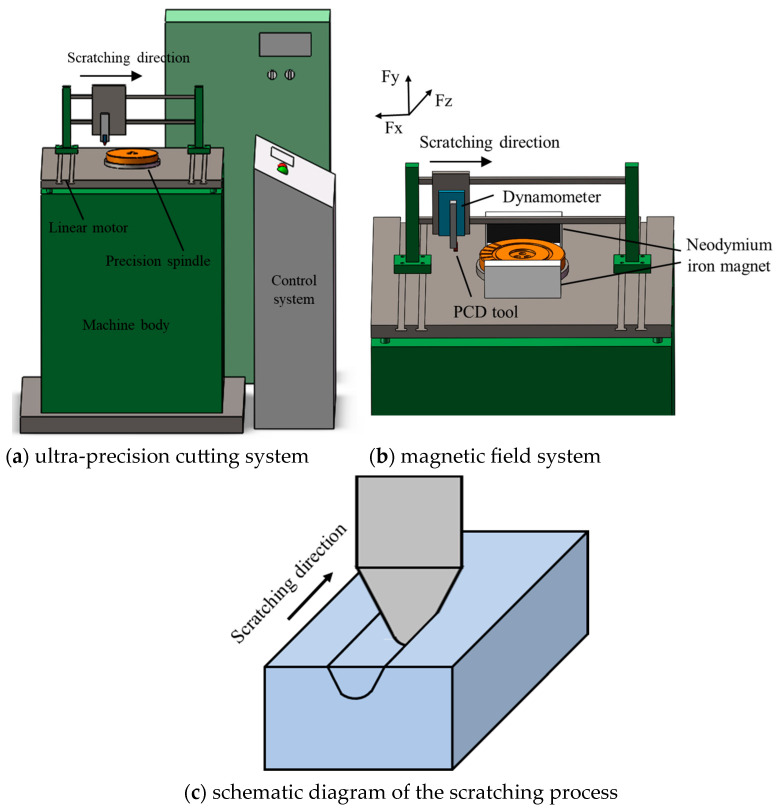
Magnetic-field-assisted scratching experiments.

The scratching tests were conducted with a self-developed magnetic-field-assisted machine tool, as exhibited in Figure 2a. The magnetic-field-assisted machine tool was composed of an ultra-precision cutting system and a magnetic-field system; the major components included a machine body, linear motor, precision spindle, control system, and magnetic field system. The ultra-precision cutting system could provide a highest rotating speed of about 12,000 rpm and a repeat position precision of ±2 μm. After adjusting the dynamic balance of the precision spindle, the system could provide an axial runout on the spindle end face of less than 1 μm to enable high-precision scratching depth. On the basis of the ultra-precision cutting system, the magnetic-field system, which was composed of two permanent magnets made of neodymium iron N35H with different polarities, was clamped on the worktable to perform magnetic-field-assisted machining, as shown in Figure 2b. The used permanent magnets were 100 × 50 × 20 mm. To prevent detrimental effects on the ultra-precision cutting system, the permanent magnets were magnetically insulated in the surrounding direction except the direction toward the workpiece using a magnetic isolation cover fixture made of 45 steel material, which could shield more than 96% of the magnetic intensity. The remained magnetic induction line was perpendicular to the scratching direction. The remanence and coercive force generated by the magnetic field system on the workpiece zone were 1.18 T and 880 KA/m.

As exhibited in Figure 2b, the scratching tests were carried out along the radial direction of the cylindrical workpiece when the turntable was locked. After finishing a scratching test, the turntable was rotated by an angle of 16° to carry out the subsequent scratching test. This ensured that the scratching tests were all completed on the same workpiece and reduced the effect caused by the use of different material batches. The single-factor experiment method was used in the scratching tests. In terms of scratching parameters, the scratching depth was set to one four levels from 4 μm to 10 μm under a constant scratching speed of 2 mm/s. The scratching speed was set to one of four levels from 2 mm/s to 8 mm/s under a constant scratching depth of 6 μm, as exhibited in Table 1. The scratching test was repeated twice in this study. In the scratching tests, the cutting force signal was recorded with a 9119AA2 dynamometer (Kistler, Winterthur, Switzerland). After the scratching test, the surface morphology was analyzed utilizing an optical surface profiler, and the chip morphology was analyzed utilizing a scanning electron microscope.

## 3. Magnetic-Field-Assisted Scratching Process

### 3.1. Magnetic Field Distribution Analysis

**Figure 3:**
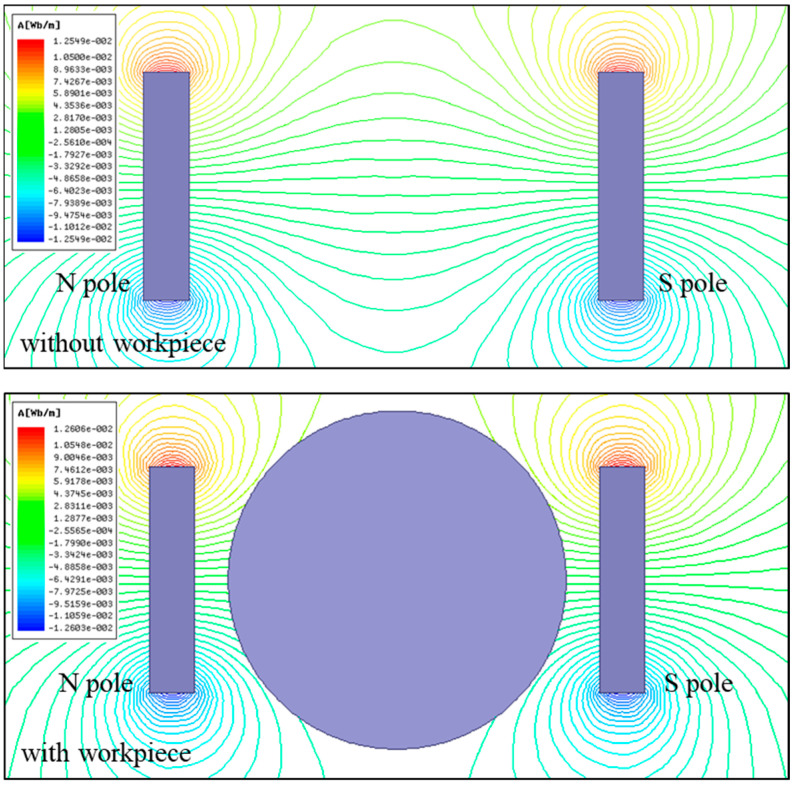
Magnetic induction line distribution.

**Figure 4:**
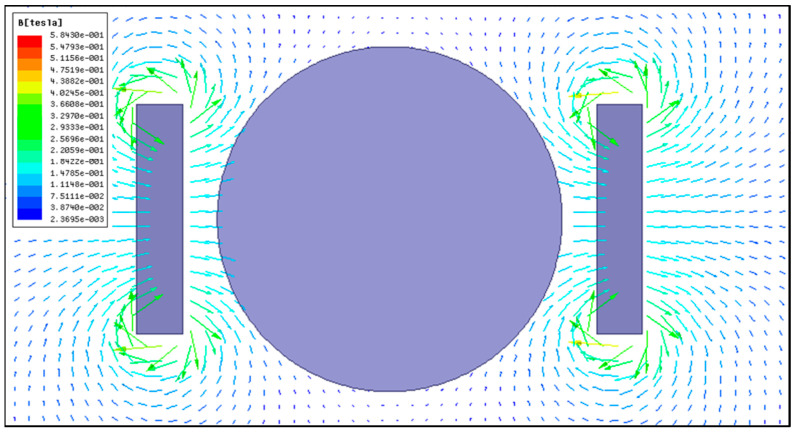
Magnetic flux density distribution.

In this work, a magnetic field system was introduced into the scratching process of single-crystal copper. The magnetic field distribution was analyzed using Ansys Maxwell 2020R1 software to investigate its effect on the scratching process. In the magnetic simulation, the iron core sizes were set according to the actual permanent magnets, and the material properties of neodymium iron N35H were as listed in Table 2. Figure 3 depicts the magnetic induction line distribution between the two permanent magnets. By comparison, the magnetic induction line was evenly distributed from the N pole to the S pole in the middle region between the two permanent magnets. And, the magnetic induction line did not exhibit the obvious variation after placing the single-crystal copper workpiece between two permanent magnets. This result was confirmed by the magnetic induction intensity measured using a tesla meter. This implied that the workpiece dud not affect magnetic field distribution between the two permanent magnets. The magnetic flux density distribution from the simulation is exhibited in Figure 4, showing the magnetic field distribution in the workpiece zone, where the arrow represents the direction of magnetic flux density. From the results, it was found that the generated magnetic field fully covered the workpiece and could offer uniform magnetic action in the scratching process.

### 3.2. Effect of Magnetic-Field-Assisted Scratching on Scratching Force

**Figure 5:**
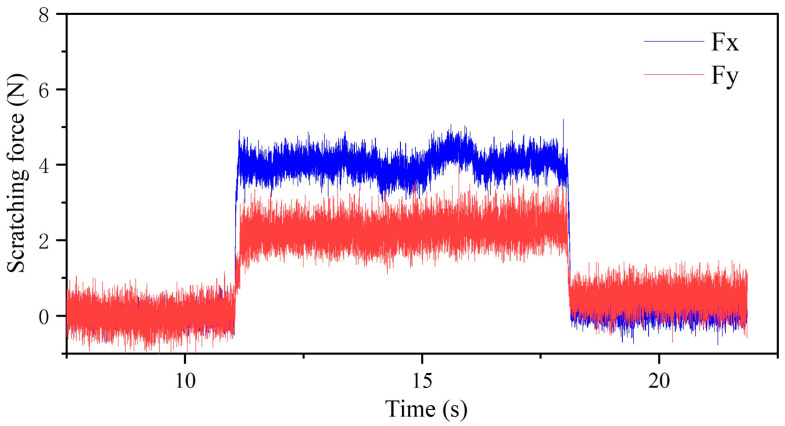
The original scratching force signal.

In the scratching test, the recorded scratching force signal was as exhibited in Figure 5. It was found that the scratching force rapidly increased during the cut-in stage and rapidly decreased during the cut-out stage. Because the scratching process is a continuous steady-state cutting process, the scratching force is very stable during the regular scratching stage. The main force components in the scratching process are the tangential force *F_t_* and the normal force *F_n_*, which exactly correspond to the recorded force components *F_x_* and *F_y_*. In this work, the statistical mean of the scratching force components at the regular scratching stage was applied for the scratching force results.

**Figure 6:**
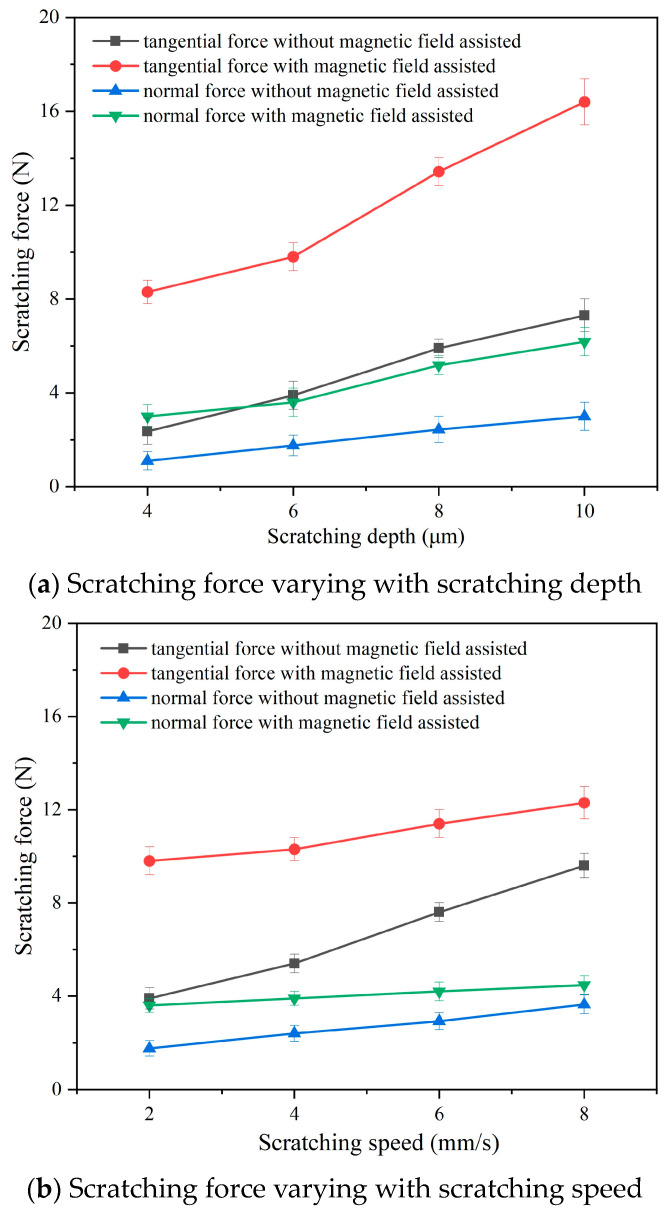
Scratching force in conventional and magnetic-field-assisted scratching.

Figure 6 exhibits the scratching forces of the conventional and magnetic-field-assisted scratching processes. Based on the results, it can be seen that the scratching forces of both conventional and magnetic-field-assisted scratching methods gradually increased with increases in the scratching depth and the scratching speed. They exhibit almost the approximate variation trend as that of the scratching parameters. It was found that the tangential force was much bigger than the normal force during the scratching process. However, the tangential force and the normal force exhibit different increase rates: the tangential force exhibits a large increase with the scratching depth and the scratching speed; the normal force exhibits only a slightly increase with the scratching depth and the scratching speed. In comparison, both the tangential and normal forces in the magnetic-field-assisted scratching were obvious larger than those with conventional scratching. The mean values of the tangential force and the normal force in the conventional and magnetic-field-assisted scratching processes were calculated and compared. Under different scratching depths, the mean values of the tangential force and the normal force in the magnetic-field-assisted scratching, respectively, increased 2.46 and 2.16 times of the force in the conventional scratching process, as exhibited in Figure 6a. Under different scratching speeds, the magnetic field action caused the mean tangential force and the normal force to increase to 1.65 and 1.51 times of the scratching forces with conventional scratching, as exhibited in Figure 6b.

**Figure 7:**
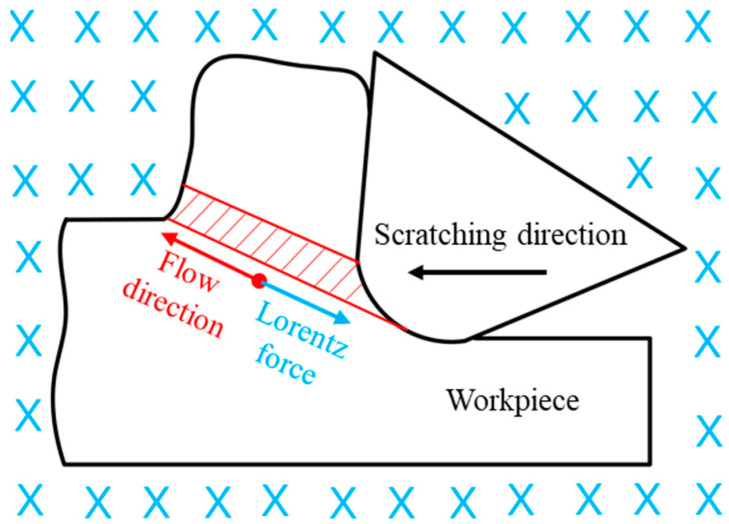
Material flow and Lorentz force in the primary deformation zone. The blue symbol represents the magnetic induction line.

The results show that magnetic-field assistance in the scratching process significantly increased the scratching force. But, the detailed reason for this phenomenon was still unexplored. During the scratching process, when the PCD tool scratches the workpiece in the cutting direction, the workpiece material experiences plastic deformation in the primary deformation zone. This plastic deformation is the plastic flow process of the workpiece material along the shear plane. The material flow direction is exhibited in Figure 7. At the same time, the magnetic induction line in the magnetic-field-assisted scratching process is perpendicular to the cutting cross-section. It is well known that single-crystal copper material is a good electric conductor, so is widely applied in various industries. Hence, the magnetic-field-assisted scratching process can be seen as a process of the electric conductor cutting magnetic induction line, as exhibited in Figure 7. When the electric conductor cutting the magnetic induction line, Lorentz force occurs in the direction contrary to the movement direction. Additional Lorentz force is superimposed on the scratching force. This likely induces the relatively larger scratching force in the magnetic-field-assisted scratching process.

### 3.3. Effect of Magnetic-Field-Assisted Scratching on Friction Coefficient

**Figure 8:**
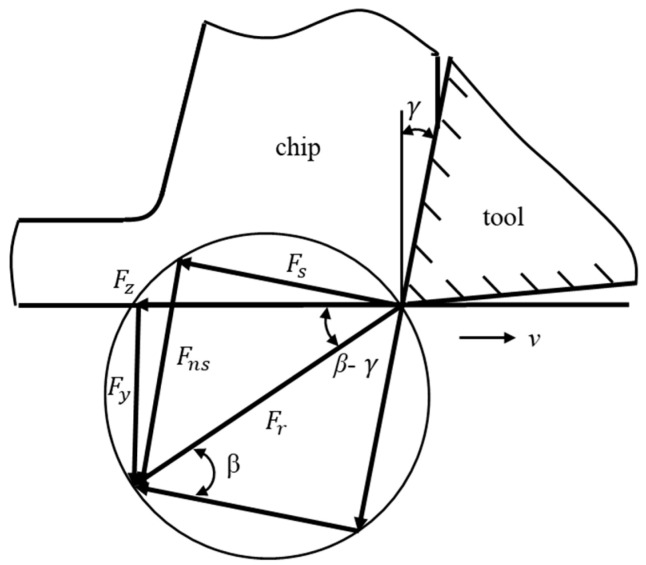
The friction angle in orthogonal cutting process.

There are two sources of cutting force in the metal cutting process: the elastic–plastic deformation of the metal material in the primary deformation zone, and the friction force in the second and third deformation zones. Among them, the friction behavior in the second deformation zone on the rake face is the main source of friction force in the metal cutting process. The friction coefficient between the chip and rake face significantly affect the cutting force components, the chip morphology, and the surface quality. As exhibited in Figure 8, the scratching process is an orthogonal cutting process; based on Merchant metal cutting theory [31], the friction angle on the rake face can be expressed as:

(1) $$\tan(\beta -\gamma )=\frac{F_{y}}{F_{z}}$$

In this formula, *β* is the friction angle on the tool’s rake face; *γ* is the tool rake angle; *F_z_* and *F_y_*, respectively, are the normal force *F_n_* and tangential force *F_t_* during the cutting process. Based on this expression, the friction coefficient tan*β* between the chip and rake face can be calculated from the cutting force results. The rake angle of the cutting tool used in the scratching tests was 0°, and then the friction coefficient could be directly calculated using the formula *μ* = *F_n_*/*F_t_*.

**Figure 9:**
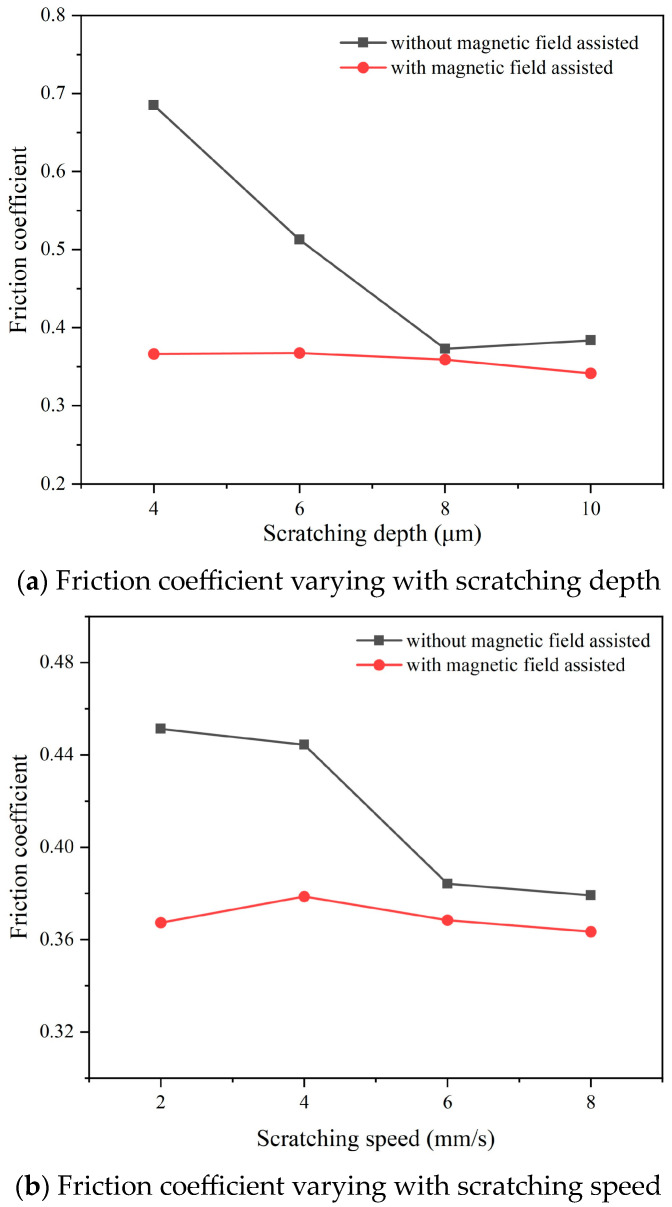
Friction coefficient of conventional and magnetic-field-assisted scratching.

Based on the scratching force results, Figure 9 exhibits the friction coefficient obtained from the conventional and magnetic-field-assisted scratching processes. The friction behavior on the rake face can be divided into two types: adhesive friction and sliding friction. The adhesive friction coefficient is much larger than the sliding friction and is the main factor that determines the friction coefficient on the rake face. In the conventional scratching process without magnetic-field assistance, it was found that the friction coefficient gradually reduced with a raise in the scratching parameters. It reduced from 0.7 to 0.4 when the scratching depth deepened from 4 μm to 10 μm. With a thicker cutting thickness, the chip deformation degree reduced; the extruding and ploughing between the chip and rake face reduced as well, thereby decreasing the adhesive friction and lowering the friction coefficient on the rake face. The friction coefficient slightly reduced from 0.45 to 0.38 when the scratching speed increased from 2 mm/s to 8 mm/s. The effect of the scratching speed on the friction coefficient was weak, being weaker than the effect of scratching depth. It was found that the friction coefficient of magnetic-field-assisted scratching was almost unchanged and stable at 0.36 under various scratching parameters. In comparison, the friction coefficients of magnetic-field-assisted scratching were all lower than those of conventional scratching. This indicated that magnetic-field-assisted scratching exhibited good friction reduction effect; the reduction in the friction coefficient averaged 19.4%. This is consistent with the findings in the literature reporting that magnetic-field-assisted cutting can improve the tribological characteristics of tool/chip contact [26,27].

### 3.4. Effect of Magnetic-Field-Assisted Scratching on Surface Morphology

**Figure 10:**
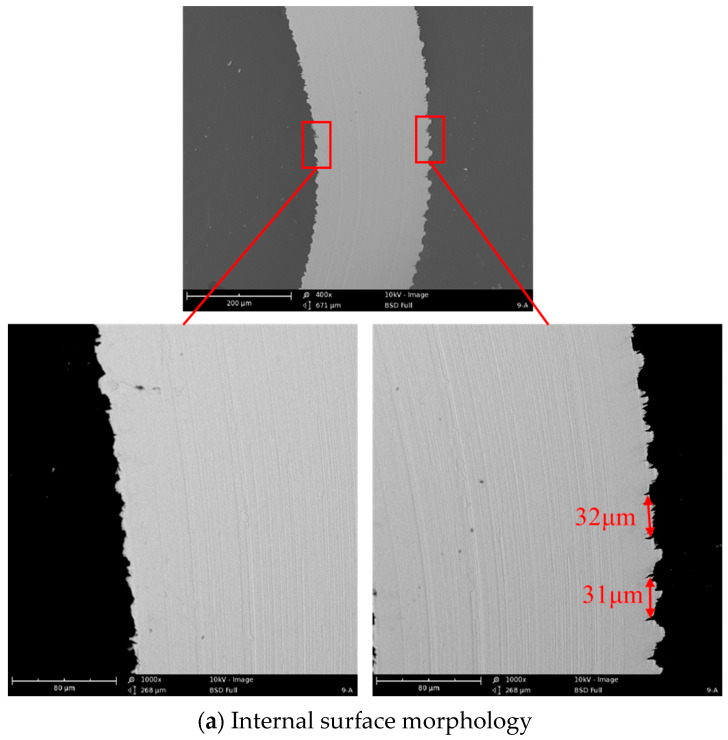

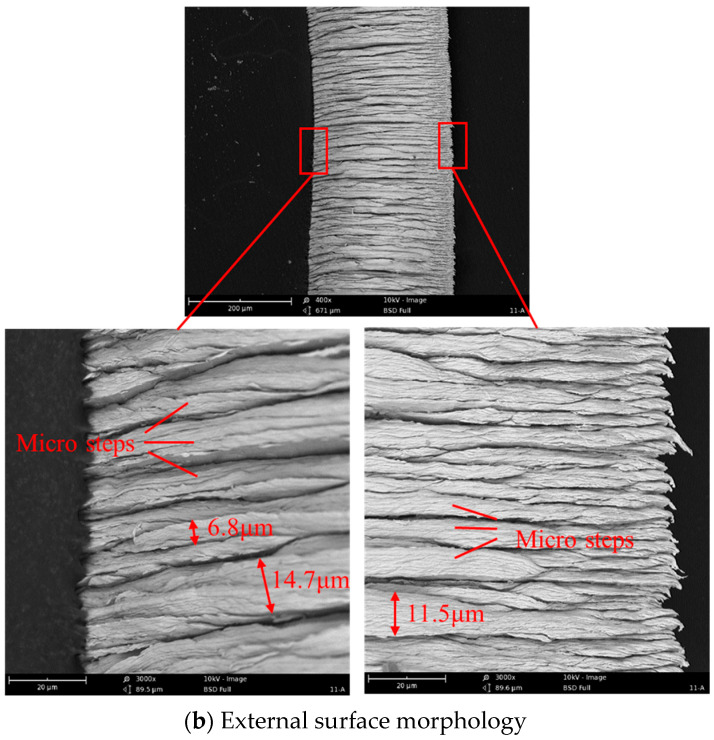
The internal and external chip surface morphology.

Chip morphology is directly related to the material’s elastic–plastic deformation and the machining quality during the cutting process. The typical morphology of the collected chips is exhibited in Figure 10. It was found that the generated chips presented a flattened shape with serrated morphology on both side edges. The spacing between serrations on the side edge was about 30 μm. This indicated that the chip encountered severe extrusion action and was seriously compressed during the cutting process. The internal surface morphology of the collected chips was very smooth with some microscratches. The internal surface of the chip experienced friction with the tool rake face; a smooth surface morphology formed via serious friction and tension behaviors. However, the external surface morphology of the chip was very rough with many protruded microsteps. These protruded microsteps formed via the plastic shear slipping of the workpiece material along the slip plane in the crystal in the primary deformation zone. A protruded microstep is a shear slip layer, and these microsteps are stacked in layers of these shear slip layers. From the results, the thickness of these protruded microsteps was uneven, changing from 6.8 μm to 14.7 μm. This indicated that the thickness of these shear slip layers changed during the cutting process.

**Figure 11:**
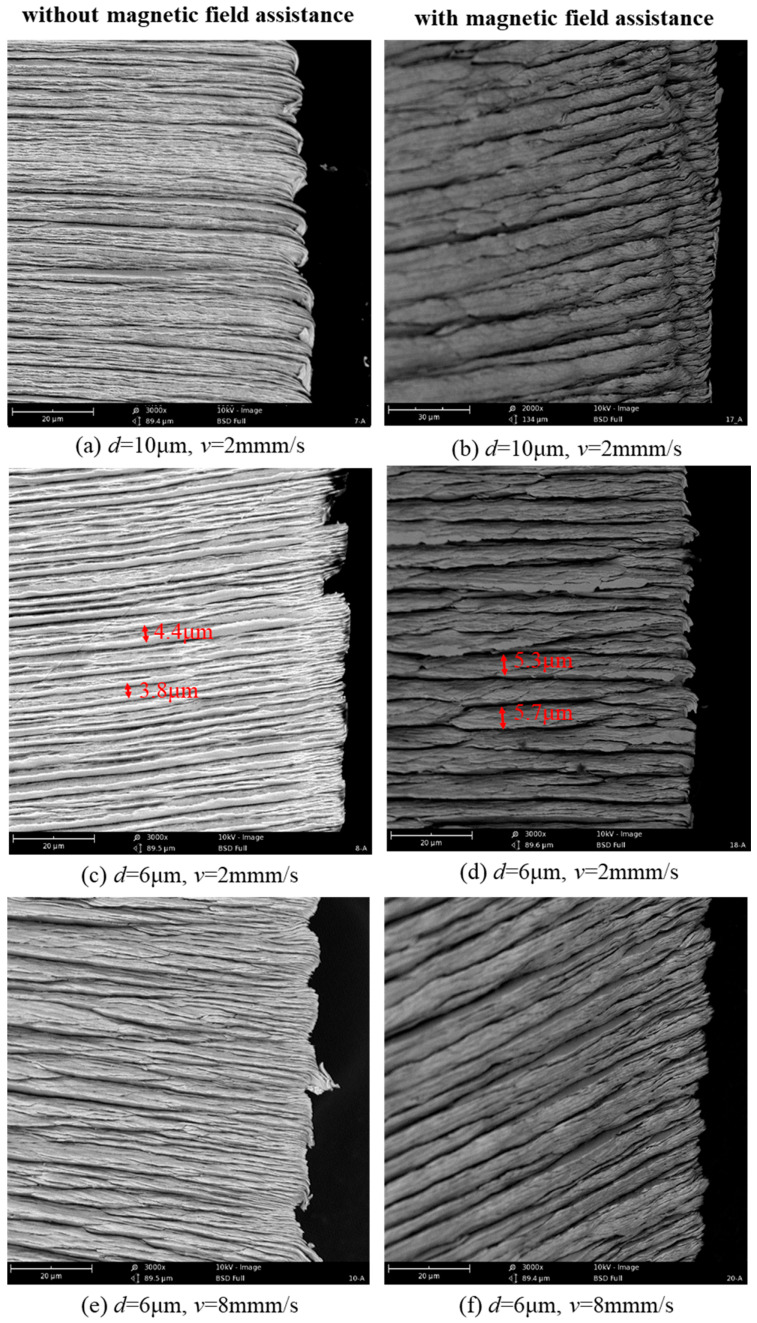
Chip morphology produced with conventional and magnetic-field-assisted scratching.

Figure 11 exhibits the external morphology of a chip in the conventional and magnetic-field-assisted scratching processes under various scratching parameters. The protruded steps on the external surface of the chip can reflect the chip deformation degree during the cutting process. It was seen that these protruded steps on the chip surface in the magnetic-field-assisted scratching were thicker but had a lower height than those of the conventional scratching process. With the scratching depth was 6 μm and the scratching speed was 2 mm/s, the thicknesses of these protruded steps in the conventional and magnetic-field-assisted scratching were about 4.1 μm and 5.5 μm, respectively. Simultaneously, it was found that there were many extended extrusion sheets between different microsteps on the chip surface with conventional scratching. This implied severe extrusion between the different shear slip layers and reflected a larger degree of chip deformation during the cutting process. These extended extrusion sheets were reduced on the chip surface with magnetic-field-assisted scratching. This indicated that magnetic field scratching is conducive to decreasing the degree of chip deformation during the metal cutting process, which is consistent to the findings in the existing literatures that the presence of a magnetic field during the cutting process can promote the dislocation slip of metal materials to affect the cutting mechanisms [23,24].

**Figure 12:**
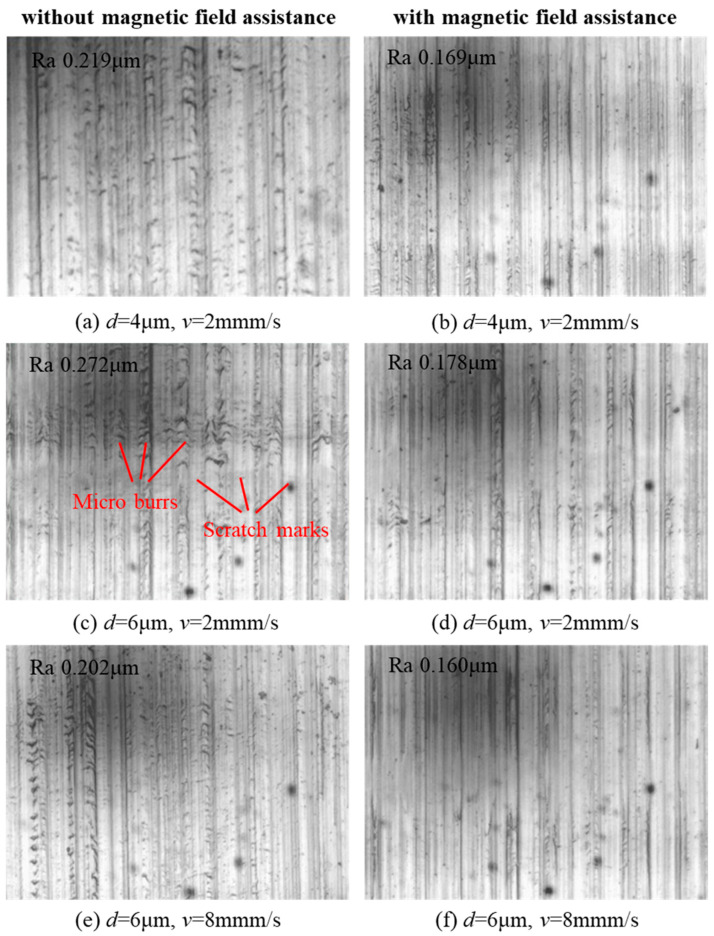
Surface morphology produced with conventional and magnetic-field-assisted scratching.

Figure 12 exhibits the bottom surface morphology of the scratched groove obtained with the conventional and magnetic-field-assisted scratching process for different scratching parameters. Scratch marks and microburrs were observed on the scratched groove surface, which were the main surface defects. The scratch marks were similarly distributed on both groove surfaces produced with the conventional and magnetic-field-assisted scratching. However, the groove surface morphology produced with magnetic-field-assisted scratching exhibited fewer microburrs and better surface quality compared with those of conventional scratching. The probable reason is the lower friction coefficient and degree of chip deformation helped to reduce material plastic side flow during the cutting process, which is mainly responsible for microburrs on a machined surface [32]. According to the roughness results, the surface roughness increased with the increase in the scratching depth and decreased with the increase in the scratching speed. In comparison, the surface roughness produced with conventional scratching was larger than 0.2 μm, but it was smaller than 0.18 μm with magnetic-field-assisted scratching. Magnetic-field-assisted scratching reduced the surface roughness by an average of 26.8%. This indicates that magnetic-field-assisted scratching can effective improve machining surface quality.

## 4. Summary and Conclusions

In this study, the scratching experiments were carried out to study the magnetic-field-assisted cutting of single-crystal copper. From the results, the following conclusions were drawn:

1. A magnetic-field-assisted cutting system was developed via the integration of an ultra-precision cutting system and magnetic field devices. The scratching force produced with magnetic field scratching was more than 1.5 times larger than that produced with conventional scratching. When the magnetic induction line was perpendicular to the cutting direction, the cutting process was equivalent to the electric conductor cutting the magnetic induction line, resulting in additional Lorentz force in the cutting process. The superimposed Lorentz force increased the actual scratching force produced with magnetic-field-assisted scratching.
2. The friction coefficient of conventional scratching gradually decreased with increasing scratching depth and speed but stabilized at a low level with magnetic-field-assisted scratching. Compared with conventional scratching, the friction coefficient of magnetic-field-assisted scratching was lower by 19.4%, showing a tribological modification effect at the tool/chip contact.
3. The chip morphology produced via the scratching of single-crystal copper exhibited a serrated side shape, smooth internal side surface, and rough external surface, with many protruding microsteps. Magnetic-field-assisted scratching helped reduce the chip deformation degree, decrease microburrs on the scratched groove surface, and reduce the surface roughness by an average of 26.8%. Based on these results, the magnetic-field-assisted cutting method helps improve the surface quality produced via the precise and ultra-precise cutting of nonferromagnetic materials and has potential applications in the fields of precision and ultra-precision cutting.

## Figures and Tables

**Table 1:** The scratching parameters.

Parameter	Value
Scratching depth *d* (μm)	4, 6, 8, 10
Cutting speed *v* (mm/s)	2, 4, 6, 8

**Table 2:** Material properties of neodymium iron N35H.

Material	Magnetoconductivity	Remanence	Coercive Force
N35H	1.05 H/m	1.18 T	880 KA/m

## Data Availability

Data are contained within the article.
